# A low-kiloelectronvolt focused ion beam strategy for processing low-thermal-conductance materials with nanoampere currents

**DOI:** 10.3762/bjnano.15.97

**Published:** 2024-09-27

**Authors:** Annalena Wolff, Nico Klingner, William Thompson, Yinghong Zhou, Jinying Lin, Yin Xiao

**Affiliations:** 1 Kavli Nanoscience Institute, California Institute of Technology (Caltech), Pasadena CA91125, USA; 2 Central Analytical Research Facility, Institute for Future Environments, Queensland University of Technology (QUT), Brisbane QLD 4000, Australia; 3 Helmholtz-Zentrum Dresden-Rossendorf (HZDR), Bautzner Landstr. 400, 01328 Dresden, Germany; 4 SLAC/Stanford, San Mateo CA 94024, USA; 5 School of Dentistry, The University of Queensland Brisbane QLD 4006, Australia; 6 Department of Implantology, Affiliated Stomatological Hospital of Xiamen Medical College, Fujian, China; 7 School of Medicine and Dentistry, Griffith University, Gold Coast QLD 4222, Australia

**Keywords:** biological sample, COMSOL, focused ion beam, forward time–centered space (FTCS), heat damage, SRIM

## Abstract

Ion beam-induced heat damage in thermally low conductive specimens such as biological samples is gaining increased interest within the scientific community. This is partly due to the increased use of FIB-SEMs in biology as well as the development of complex materials, such as polymers, which need to be analyzed. The work presented here looks at the physics behind the ion beam–sample interactions and the effect of the incident ion energy (set by the acceleration voltage) on inducing increases in sample temperature and potential heat damage in thermally low conductive materials such as polymers and biological samples. The ion beam-induced heat for different ion beam currents at low acceleration voltages is calculated using Fourier’s law of heat transfer, finite element simulations, and numerical modelling results and compared to experiments. The results indicate that with lower accelerator voltages, higher ion beam currents in the nanoampere range can be used to pattern or image soft material and non-resin-embedded biological samples with increased milling speed but reduced heat damage.

## Introduction

FIB-SEMs combine a scanning electron microscope (SEM) and a focused ion beam (FIB) in a single instrument and are increasingly used to prepare cross sections and TEM lamellae of biological samples as well as of other thermally low conductive materials such as polymers [1–13]. The easily induced heat damage is increasingly being reported [3,6,14–17]. Despite the importance of this topic, there is not a large number of recent papers published looking at the underlying physics in the field of focused ion beams. A broader look at current literature on heat transfer induced by particle beams highlights that heat damage is not only problematic for FIB processing, but also presents challenges for light interactions with biological tissue [18] as well as focused electron beam-induced deposition (FEBID) [19]. The general approach to assess the beam-induced heat damage and undesired artifacts, regardless if working with ions [17], photons [18], or electrons [19], compares experiments to models based on heat transfer and to Monte Carlo or finite element simulations [17–19]. Open source programs that assess heat deposition and diffusion are readily available to assess damage in light–tissue interactions [18]. For electron beams, multidimensional models predicting electron beam-induced heating effects that lead to noticeable changes in nanostructure deposition geometry during FEBID processes have recently been published [19]. These approaches, a thorough understanding of the parameters that govern the beam-induced heat damage as well as open source software, would also be beneficial for the FIB community, especially with the increase in FIB milling of thermally low conductive materials.

Recent work assessed the ion beam-induced heat damage and how this can be limited by FIB parameter choice [16–17]. The reported results suggested that ion beam-induced heat can be minimized by using lower ion beam currents [16–17] and by reducing the beam overlap as well as blurring the beam [17]. The drawback of this approach, however, is increased processing times due to the small current (picoampere range). Other approaches that were successfully used to avoid or reduce heat damage include working at cryogenic sample temperatures, [16], short beam dwell times [16], as well as employing different scan strategies [16]. This work builds on previously reported experiments [17] and looks at the effect of the ion energy on the ion beam-induced sample heating to maintain nanoampere beam currents and fast processing times. Suitable ion beam parameters for lower-energy ions (5 keV) are assessed, and the results are compared to the conventionally used 30 keV ions.

The interactions of 5 keV gallium ions and the effect on heat accumulation due to ion impacts with the sample (skin) were investigated using the Monte Carlo simulation program SRIM [20], the program COMSOL (finite element analysis platform), and a numerical analysis using the forward time–centered space method to solve the 3D heat equation. This approach is discussed in detail elsewhere [17]. The results are experimentally tested by milling a TEM lamella and assessing the ion beam-induced heat damage in collagen.

Purified collagen was selected as the experimental test material for two main reasons. First, it is the principal component of skin, for which well-documented literature values are readily available [21]. Second, collagen’s fibrillar structure, visible by microscopy, is denatured by heat to give gelatin that lacks any fixed structure [22–23], making heat damage easily recognizable.

Despite the focus on Ga ions impacting in skin (simulations) and collagen (experimental), the broader results presented here are true for any type of instrument and ion species, which includes gallium FIBs, plasma FIBs, helium ion microscopy FIBs, as well as low-temperature ion source and magneto-optical trap ion source FIBs.

## Results and Discussion

### SRIM simulations

**Figure 1:**
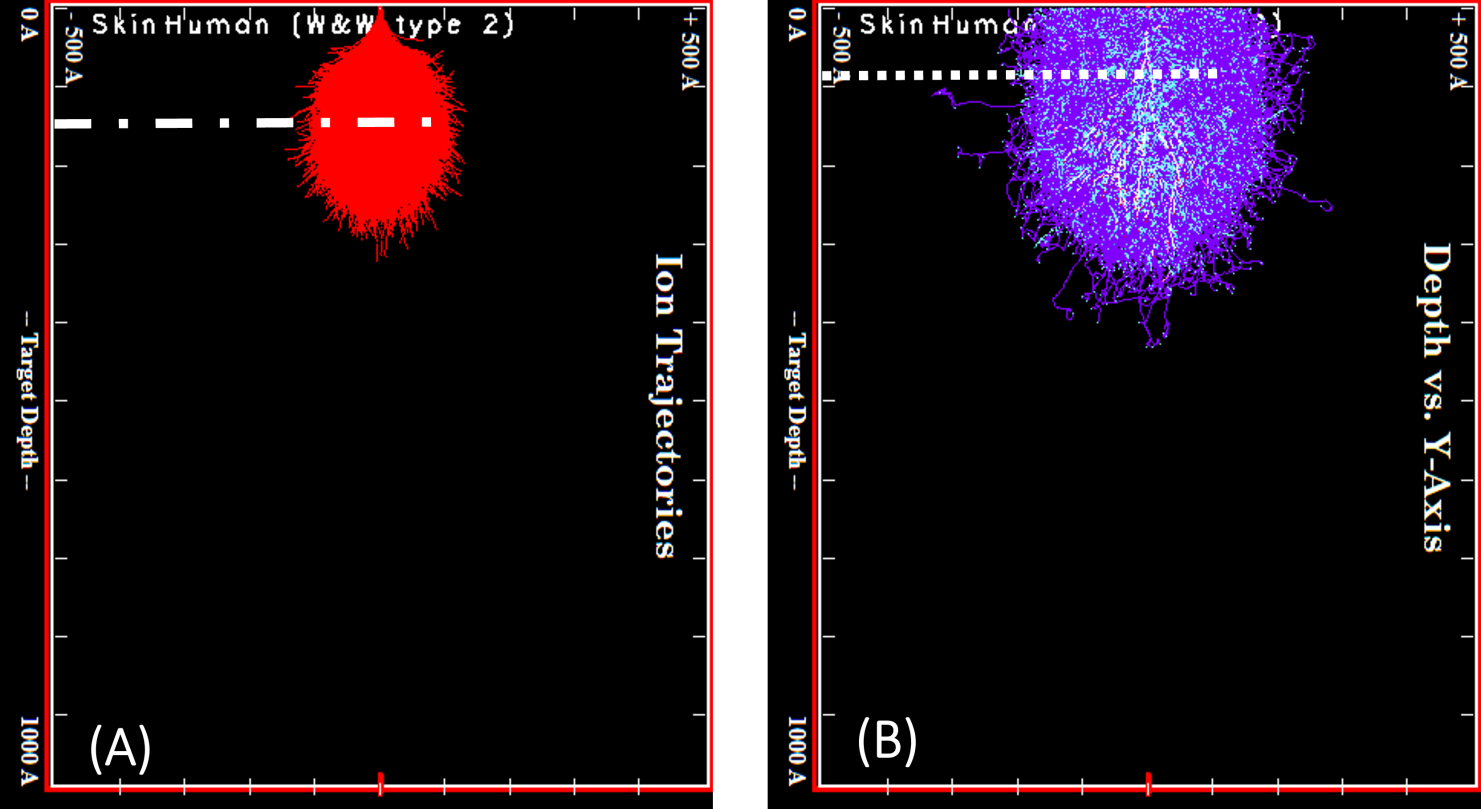
(A) Ion trajectories for 5 keV gallium ions (Ga^+^) in human skin. SRIM/TRIM assumes a beam with a single impact point. The projected range *R*_proj_ is marked as the dashed line in the ion trajectories plot. The peak phonon and ionization positions are marked in the collision cascade plots (B) as a dotted line.

SRIM simulations were carried out to evaluate the interactions of 5 keV gallium ions with skin. The results are displayed in Figure 1. The projected range is highlighted in the ion trajectories plot in Figure 1A. The recoiling sample atoms in the collision cascade create the majority of phonons and ionization (see cascade plots in Figure 1B) and, therefore, need to be considered when evaluating the heat flux into skin (refer to Table 1). The simulated energy losses, given in Table 1, show that the ions lose the majority of their energy (>90%) to phonons and ionization at 5 keV incident energy. This result is in good agreement with the literature [13,15]. The total amount of energy loss to phonons and ionization and, therefore, heat is significantly (by a factor of six) lower for 5 keV ions than for 30 keV ion [17] as the incident ions have less initial energy to lose. The sputtering yield is reduced (by a factor of two) for 5 keV ions in comparison to 30 keV ions [17]. This means that more 5 keV ions are required to remove the same volume than higher-energy ions. The stopping power is 32% lower, which corresponds to the deposited energy per ion and depth interval. This means that, per implanted ion, around 32% less energy per volume will be deposited. Taking this into account, the SRIM simulations suggest that FIB processing should be three times faster when using a lower acceleration voltage of 5 kV instead of 30 kV.

**Table 1:** SRIM calculation parameters showing the total energy loss to phonons and ionization per incident gallium ion in skin.

Energy [keV]	5	30
Energy losses to phonons per ion [keV]	3.3	17.1
Energy losses to ionization per ion [keV]	1.4	11.8
Energy losses to ionization and phonons per ion [keV]	4.7	28.9
Ion range [Å]	154	467
Sputtering yield per incident ion	1.4	2.9
Stopping power [eV·Å^−1^]	56.5	83.8

### COMSOL simulations

**Figure 2:**
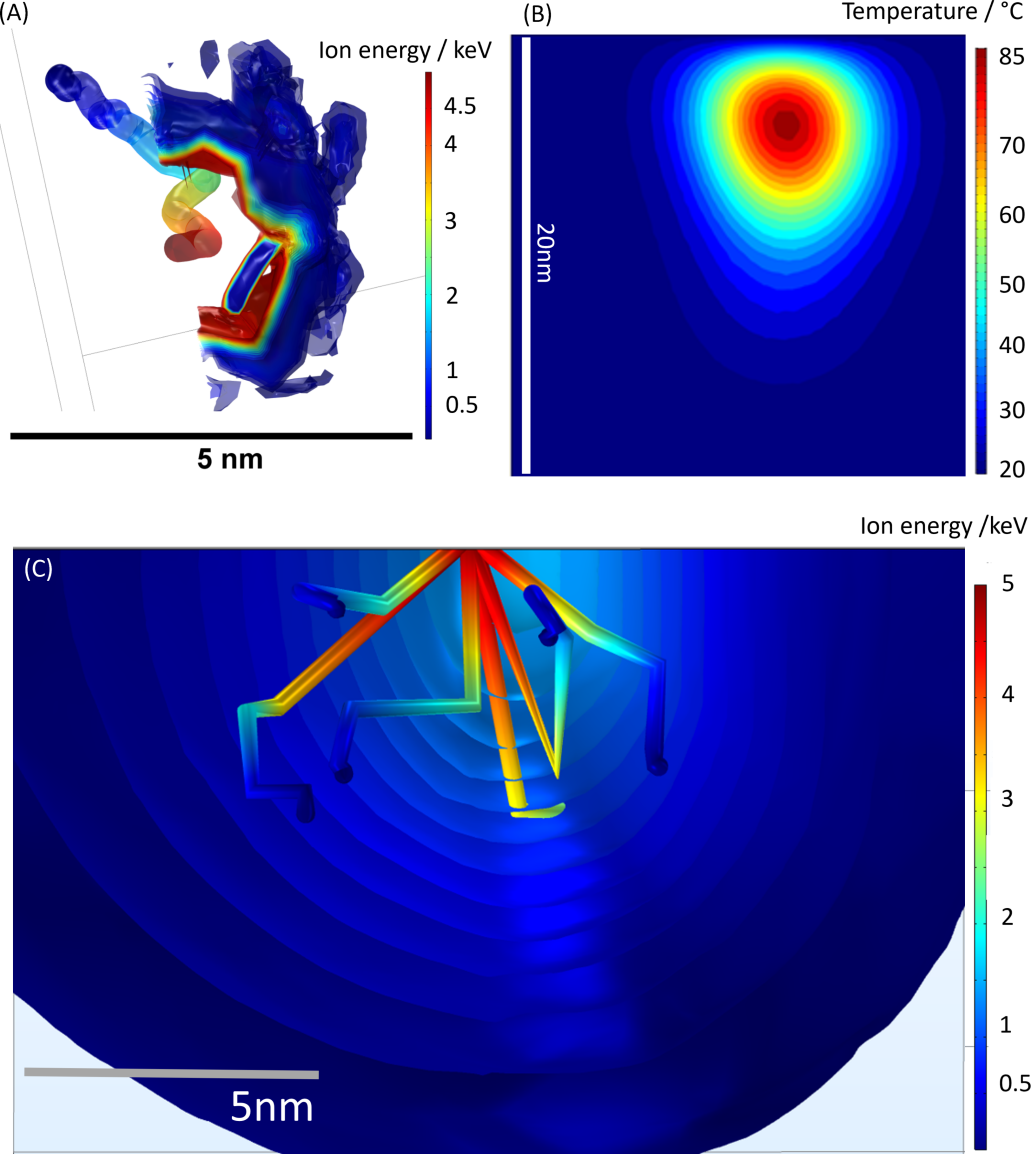
COMSOL simulations of 5 keV gallium ions (Ga^+^) interacting with collagen. Top row: Single ion impact simulations. (A) Top view of a single 5 keV gallium ion trajectory in collagen and (B) the single ion vertical mid-plane temperature isosurface. (C) Trajectories and temperature isosurfaces for six consecutive ion impacts with a 1 nA ion beam current for 5 keV Ga^+^ in collagen.

The heat induced by a single gallium ion impacting collagen as well as multiple ion impacts were studied using COMSOL simulations. Figure 2A,B shows the top view of a single 5 keV ion track. The simulation suggests that any irreversible sample damage that may occur around each ion track is contained well within 5 nm. The surface area around each ion track can be sputtered away during the milling process. Irreversible heat damage from the individual ion impact can be neglected if heat damage does not extend into the surrounding tissue.

To qualitatively assess the effect of multiple ion impacts within a short timeframe, six ion impacts for 5 keV ions were simulated. For a 1 nA beam, ions hit the sample on average every 160 ps. A scanned beam will generally not have moved away from any impact site before 100 ns have elapsed. These 100 ns are currently the typical minimal dwell time for most FIB machines. Consequently, for a 1 nA beam, approximately 625 ions will impact the same scan point before the focused ion beam spot has been moved one DAC step further. For a system with 1 nm placement accuracy, the sample, if not cooled to room temperature before those 160 ps have elapsed, will be heated continuously at this location. As long as minimal dwell times cannot be shortened below 1 ns, there will be heat accumulation within a single scan point when using nanoampere beam currents and nanometer spot sizes.

For 5 keV ions, the cooling time constant was determined from the COMSOL simulations to be around 170 ps, which is similar to the time between ion impacts for nanoampere ion beam currents and significantly shorter than the 1 ns cool down time constant for 30 keV ions [17]. The results indicate that, in the case of 5 keV ions, there might be sufficient time for the induced heat to dissipate between ion impacts or to at least allow for a significant reduction in induced heat before a subsequent ion impact. At 160 ps, just before the arrival of the next ion in a 1 nA beam, the central region below the impact point of that ion, has cooled down to 80 °C. Figure 2 shows the contour plots of the mid-plane collagen sample temperature at *t* = 160 ps for a 5 keV ion.

The ion cascade cumulative and adjacent heating terms were omitted in these simulations, but the ions’ electronic stopping power heating term was included. As isosurfaces beyond the ion tracks cannot be observed for the 5 keV ions, the cumulative heat damage does not seem to substantially occur for these lower incident ion energies (Figure 2C).

### 3D numerical heat transfer approach

**Figure 3:**
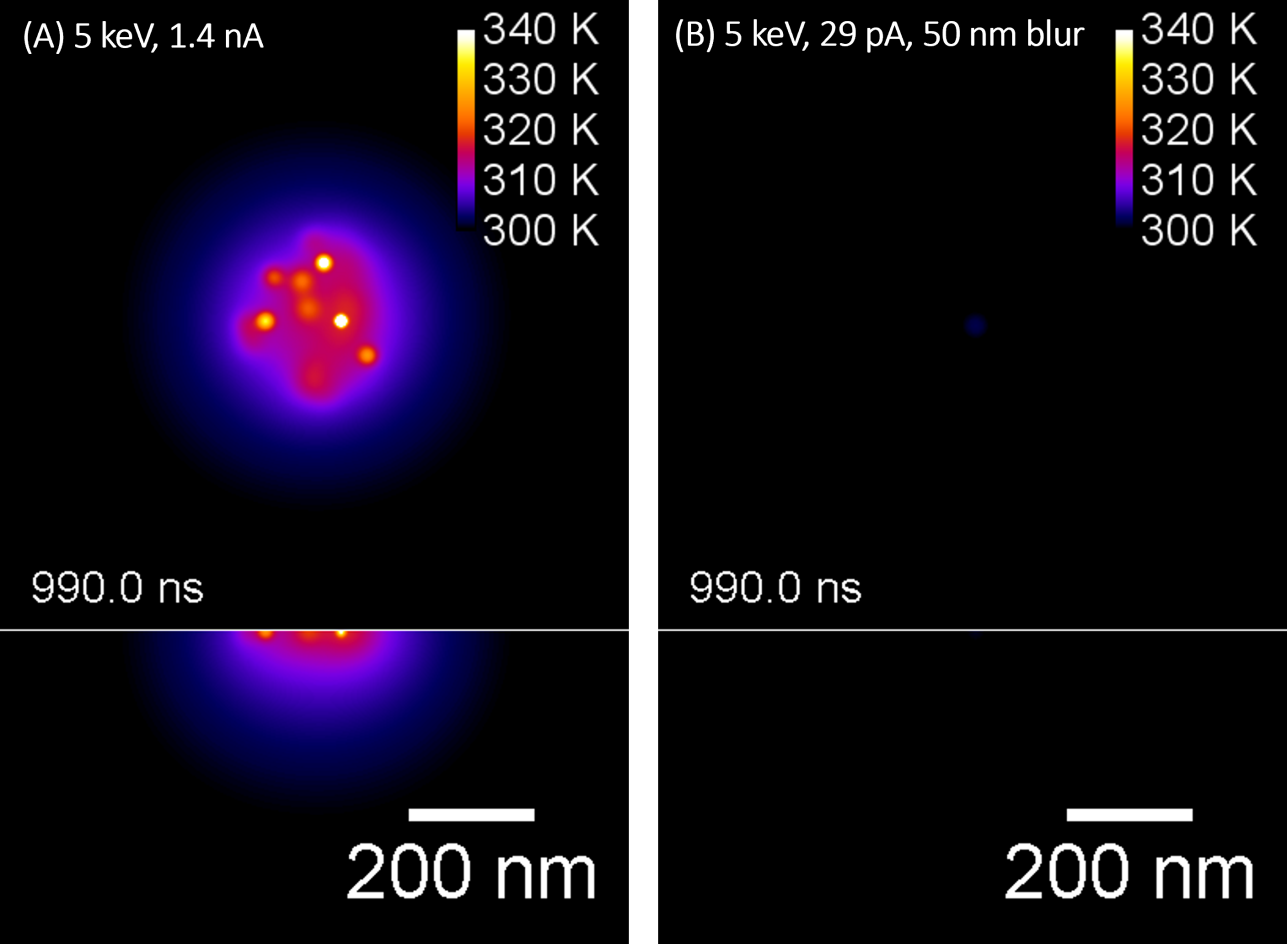
Illustration of the numerically solved three-dimensional heat equation for 5 keV Ga^+^ ions in collagen for different ion beam currents, (A) 1.4 nA and (B) 29 pA, assuming a 50 nm Gaussian beam profile. The surface temperature (top view) surface and the temperature of a cross section (side view, below) through the middle of the ion beam spot have been plotted after an irradiation time of 990 ns. The color scales are fixed to 340 K max for better comparison.

A 3D numerical heat transfer approach was used to include the effect of multiple ion impacts. A time-dependent heat equation was used to assess the effects of global and local heat damage for 5 keV Ga ions when using picoampere and nanoampere ion beam currents. The surface and a cross section through the middle of the ion beam spot for a simulated sample volume of 600 nm × 600 nm × 400 nm after an irradiation time of 990.0 ns is shown in Figure 3.

The simulations for 5 keV in the nanoampere beam current range (Figure 3A) and picoampere beam current range (Figure 3B) show a significantly lower temperature rise compared to the earlier reported 30 keV ions [16]. Only a slight temperature increase of less than 35 K to approximately 60 °C can be observed when simulating 5 keV ions with 1.4 nA beam current. Furthermore, the induced increase in temperature is just below the denaturation temperature for collagen of 65 °C [24]. The numerical modelling results suggest that the ion beam-induced temperature as well as the area of elevated temperature (Figure 3B, dark blue area) can be reduced further (in this case well below 10 K of temperature change) by using 5 keV ions in the picoampere beam current range. The simulation results suggest that using lower ion energies such as 5 keV would allow one to FIB-process many biological as well as soft materials with beam currents from the picoampere to the nanoampere ion beam current range.

### Proposed model to estimate changes in sample temperature

The previously reported model [16]


[1] $$\Delta T[\text{K}]=\frac{\left(\Delta E_{\text{el}}\;[\text{J}]+\Delta E_{\text{phonon}}\;[\text{J}]\right)*\text{N}}{t\;[\text{s}]*A\;[{\text{m}}^{2}]}*\frac{R_{\text{proj}}\;[\text{m}]}{k\;\left[\frac{\text{W}}{\text{mK}}\right]}$$

is used to estimate the ion beam-induced changes in sample temperatures. A detailed description of the model is given in the previous publication [17].

The derived Equation 1 qualitatively suggests that reducing the ion energy (set by the acceleration voltage) directly allows the ion beam-induced temperature increases per scan point to be minimized. Materials with a low thermal conductivity such as polymers or biological samples, for example, skin with a thermal conductivity of 0.29 W·m^−1^·K^−1^ [21], will show excessive increases in temperature (Δ*T*_skin_ = 2500 K according to Equation 1) when using conventional ion beam parameters such as 30 kV acceleration voltage with nanoampere range currents [17].

A low-ion-energy approach using 5 keV gallium ions while using the same ion beam current of 1.4 nA is expected to lead to a locally increase in temperature of Δ*T*_skin_ = 7.5 K. The model predicts that much less heat damage will occur when using 5 keV energy ions despite using nanoampere range currents. This is in good agreement with the numerical modelling results and COMSOL simulations. Table 2 below shows the calculated results for 5 keV ions for various nanoampere and picoampere range beam currents with and without blur. The modelling results suggest that the increase in sample temperature can be further minimized by reducing the beam current and blurring the beam. This behavior matches the previously reported behavior for 30 keV ions [17]. Even though this is not expected to be necessary in the case of processing collagen, further reducing the ion beam-induced heating by lowering the beam current and blurring the beam might be required for some polymers or biological samples that have a lower thermal conductivity than skin.

**Table 2:** Summary and comparison of ion beam parameters, the predicted increase in temperature, and experimentally observed heat damage in Figure 4 and Figure 5 for 5 keV and 30 keV gallium ions impacting on skin.

Figure Nr.	Ion beam setting	Dose [ions·cm^−2^]	Heat flux [µW]	Ion impacts per ns	Dose rate [ions·µs^−1^·nm^−2^]	Δ*T*_skin_ [°C]	Observed heat damage

4A	30 keV, 1 nA, 20% overlap, 200 nm blur	1.4 × 10^13^	30	6	0.2	114	yes
4B	5 keV, 1.4 nA, 50% overlap	1.8 × 10^13^	6.6	9	0.18	7.5	no
5A	5 keV, 29 pA, 20% overlap, 50 nm blur	9.2 × 10^12^	0.14	0.2	0.09	3.8	no
5B	5 keV, 70 pA, 20% overlap, 50 nm blur	2.2 × 10^13^	0.33	0.4	0.22	9	no
5C	5 keV, 0.12 nA, 50% overlap, 93 nm diameter	1.1 × 10^13^	0.56	0.7	0.11	4.5	no
5D	5 keV, 29 pA, 20% overlap, 200 nm blur	5.8 × 10^11^	0.14	0.2	0.006	0.2	no
5E	5 keV, 70 pA, 20% overlap, 200 nm blur	1.4 × 10^12^	0.33	0.4	0.01	0.6	no
5F	5 keV, 0.12 nA, 20% overlap, 200 nm blur	2.4 × 10^12^	0.56	0.7	0.02	1	no

### Comparison of simulations and experimental data

**Figure 4:**
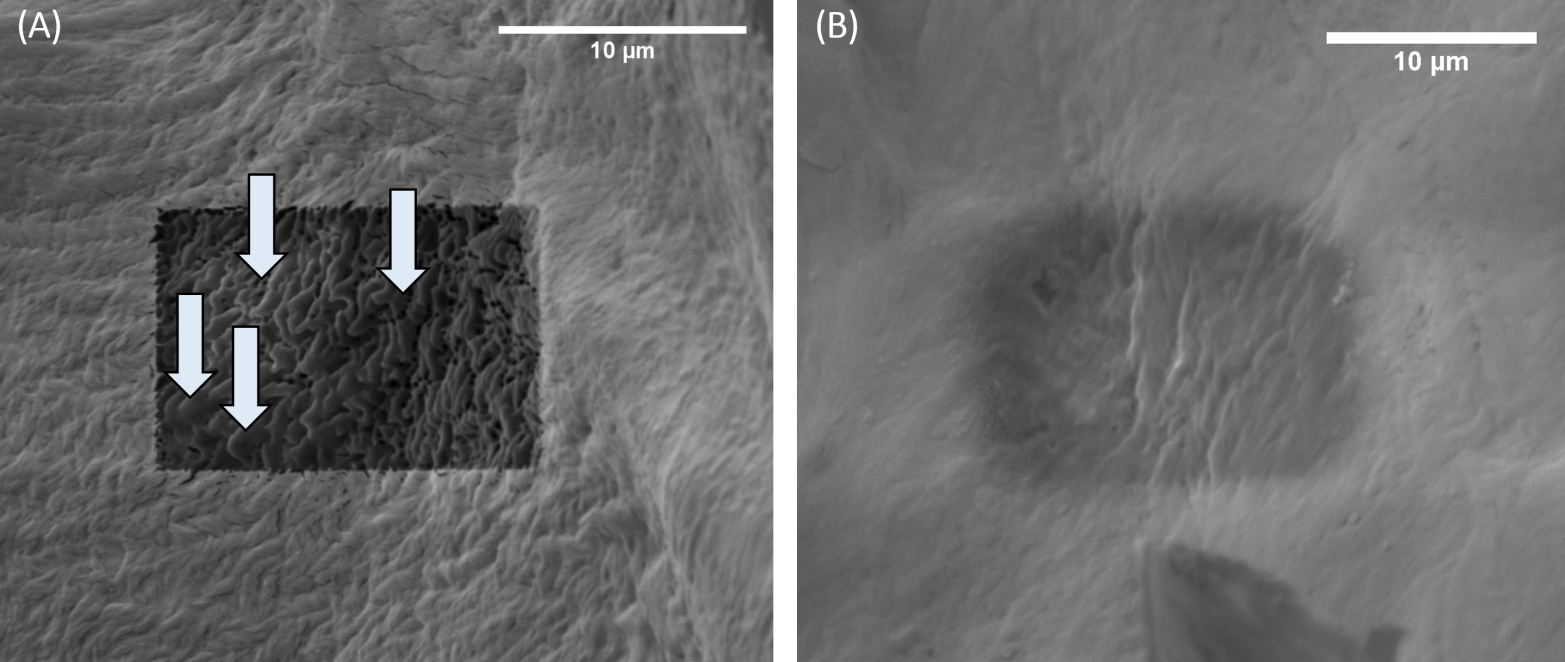
SEM micrographs showing cross sections that were cut into collagen using (A) 30 keV gallium ions with 1 nA beam current and 1.4 × 10^13^ ions·cm^−2^ and (B) 5 keV gallium ions with 1.4 nA beam current and 1.8 × 10^13^ ions·cm^−2^. Unnaturally smooth areas within the cross section cut using 30 keV cannot be observed in the cross section that was cut with 5 keV.

**Figure 5:**
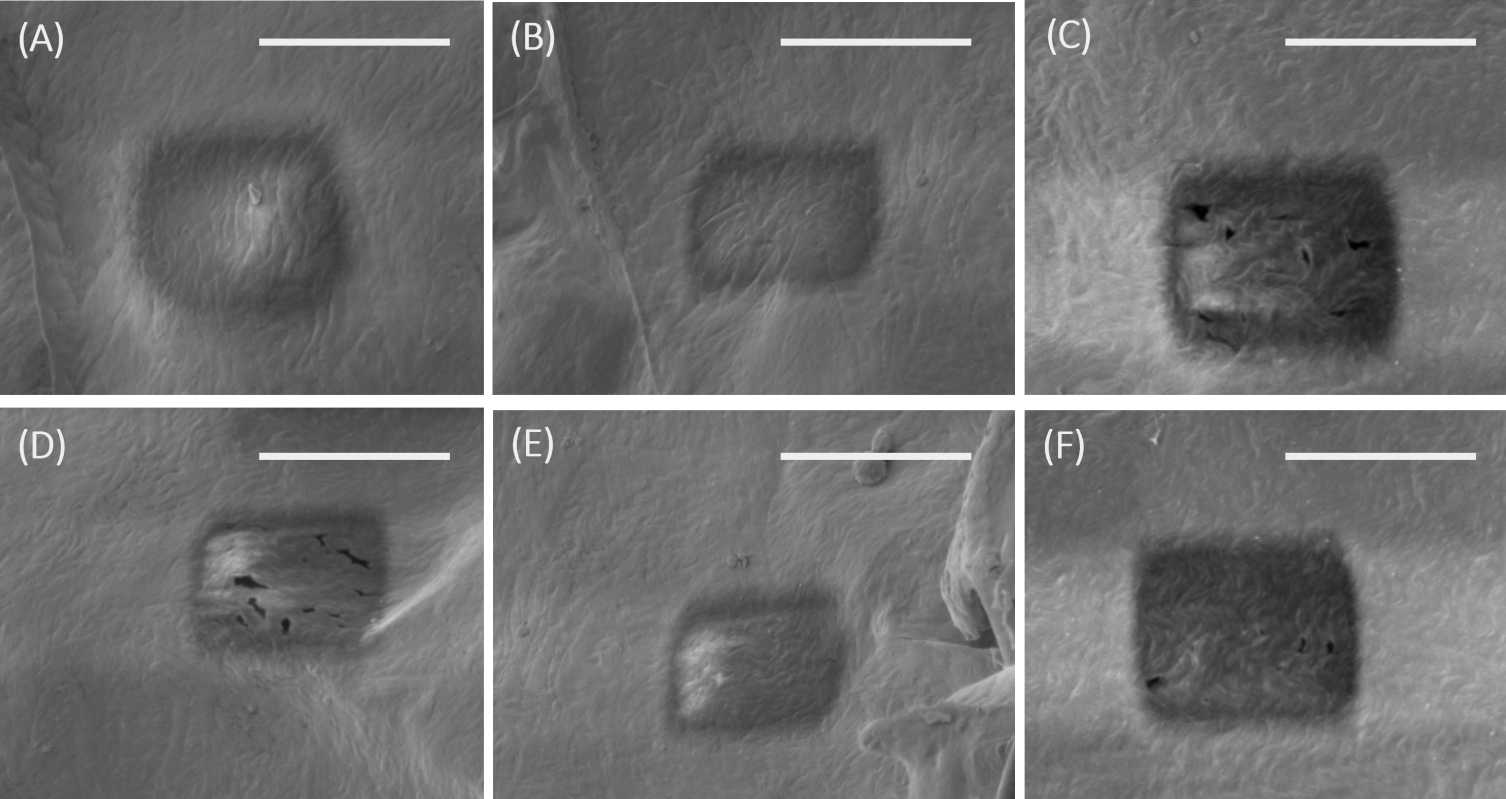
SEM micrographs showing cross sections that were cut into collagen using 5 keV and different ion beam settings: (A) 29 pA, 20% overlap, 50 nm blur; (B) 70 pA, 20% overlap, 50 nm blur; (C) 0.12 nA, 50% overlap; (D) 29 pA, 20% overlap, 200 nm blur; (E) 70 pA, 20% overlap, 200 nm blur; and (F) 0.12 nA, 20% overlap, 200 nm blur. The scale bars correspond to 10 µm.

Cross sections were cut into collagen using 5 keV energy Ga ions to evaluate the results from the simulations and the proposed model. One cross section was cut with an acceleration voltage of 30 kV, beam current of 1 nA, 200 nm blur, and 20% overlap to assess the heat damage when using nanoampere beam currents. The unnaturally smooth surface areas, visible in the cross sections in the SEM image in Figure 4A, indicate that heat damage occurs when using higher ion energies and nanoampere currents for collagen, even when blurring the beam and reducing the overlap. This result agrees well with the numerical modelling results as well as previously reported results [17]. The heat damage is not visible when using a reduced ion energy of 5 keV despite using nanoampere currents and no blur or reduced overlap (Figure 4B). Further cross sections were cut using 5 keV ions with various picoampere range currents (see Figure 5) with and without blur and reduced overlap. Heat damage cannot be observed for any of the cross sections that were cut into collagen using 5 keV ions. The results match the predicted outcomes from the numerical modelling and the model.

Reducing the ion beam energy reduces the amount of energy that is transformed into heat for each ion impact per unit of time. In addition to a lower energy loss to heat within the sample when reducing the ion energy, the final beam diameter is increased for lower energies in comparison to higher energies. The area underneath the ion beam is increased; therefore, the smaller resulting local ion dose and dose rate both play a significant role in additionally reducing heat damage. This reduces the probability by a factor of *r*^2^, with *r* being the ion beam radius, of an ion impact in close proximity to the previous impact and reduces any potential accumulative increase in local temperature.

Being able to use a higher ion current in the nanoampere range in comparison with the previously suggested heat-reduced approach (where the beam current was reduced) addresses the issue of increased patterning times and cross sections with unfeasibly large area.

**Figure 6:**
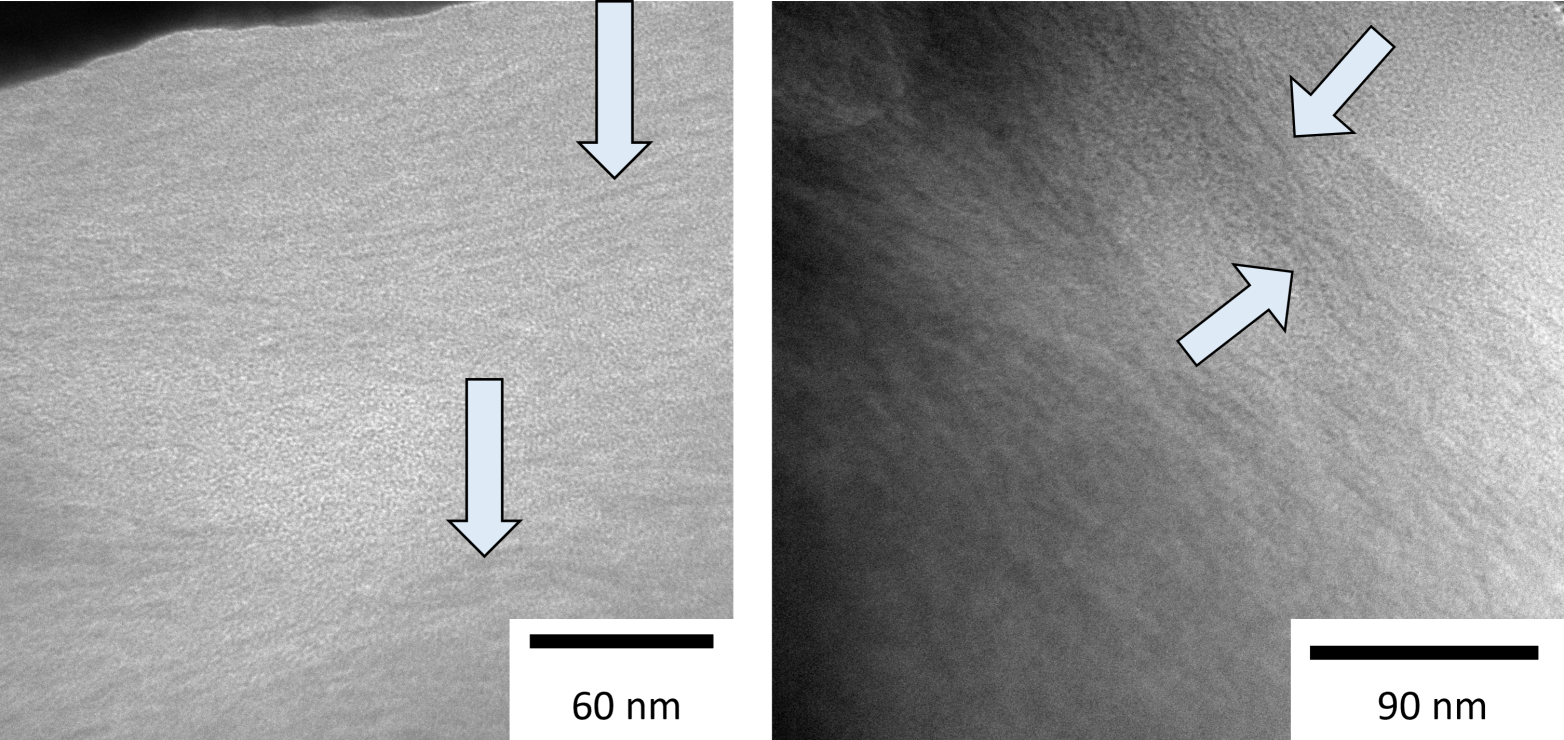
TEM micrographs showing cross sections of collagen prepared with 5 keV gallium ions. The collagen fibers are visible in the TEM lamella prepared with currents in the nanoampere range.

To further verify that no heat damage occurs, a TEM lamella was prepared using the lower-ion-energy approach (5 keV, nanoampere beam currents). The result is shown in Figure 6. Collagen fibers can be observed for the lower-energy approach, indicating that heat damage was minimized. The observed structure appears the same as for the previously reported reduced-heat approach and microtome results of the sample [17]. The experimental results obtained from the cross-sectioning and the TEM lamella comparison are in good agreement with the predictions from the simulations and the proposed model. Thermally low conductive materials can be processed at room temperature with nanoampere currents in the FIB when the ion energy is lowered.

Cryo FIB preparation has become a key enabling technology in recent years and reports on successful cryo FIB preparation of biological specimens at higher ion energies can be found throughout the literature [25–26]. Heating has not been observed when working with frozen hydrated samples. The thermal conductivity of ice is 2–3 W·m^−1^·K^−1^ [27]. FIB-induced heat damage would not be expected for cryo FIB processes, since the thermal conductivity of cryo samples is orders of magnitude higher than that of the thermally low conductive materials used in this report.

## Conclusion

The effects of acceleration voltage and ion energy on the heat damage during FIB processing was assessed using Monte Carlo simulations, finite element simulations, time-dependent numerical modelling approaches, a model based on Fourier’s law of conductive heat transfer, as well as cross-sectioning and TEM lamella preparation. Heat damage that extends into surrounding tissues could be avoided while using high currents when the sample was processed using lower ion energies (such as 5 keV).

By reducing the ion energy from 30 to 5 keV, the loss to heat can be reduced by a factor of six, while the sputtering speed is only reduced by a factor of two. The reduction in energy allows the operator to use nanoampere beam currents, which addresses the drawback of the previously reported approach of unfeasibly long patterning times. The results suggest that an additional reduction in ion beam current and applying blur and reduced overlap, when using low ion energies, can further reduce the induced heat. Using that combination might be required for processing materials exhibiting an even lower thermal conductivity than skin.

The theoretical work presented here assumes an isotropic and homogenous and flat specimen. Samples are generally more complex, exhibiting different sample topographies as well as material anisotropies. These factors are likely to play a crucial role, and future work should be carried out to better understand the effect of complex heat transfer mechanisms. In addition, other FIB processing parameters, such as scan strategies, should be incorporated and the effect evaluated in future work to further reduce the local heating, as demonstrated by Schmied et al. in an earlier study [16]. Schmied et al. showed that the dwell time plays a significant role in reducing local heating [16]. The effect of the ion dose rate/energy converted to heat per time is, thus, an important parameter and should be carefully investigated in future experiments.

In addition, the effect of the ion incidence angle and associated changes in interaction volume shape as well as heat generation need to be investigated further. The ion beam usually hits the sample at glancing angles during TEM lamella preparation and cross-sectioning, which leads to anisotropic changes in the interaction volume. Whilst the heat generation here was minimized, with irreversible heat damage limited to 5 nm, elevated temperatures (<20 K difference) can be observed to about 10 nm beyond the ion impact point. This is a significant distance when compared to a prepared TEM lamella, which has a typical thickness of 50 to 100 nm. Whilst this temperature does not cause any damage to this sample, elevated temperatures beyond the impact point can potentially cause heat damage, especially when working with materials with a lower thermal conductivity or a lower melting point than collagen. Future experiments should carefully evaluate if changes to the ion incidence angle can lead to a further reduction in ion beam-induced heat damage. The effect of the different interaction volume sizes and shapes for various ion species should be studied to better understand where the proposed 1D model works and where a 3D model needs to be applied. Mutunga et al. showed that the 1D model overestimates the induced temperature in comparison to the 3D model [19]. A careful evaluation needs to be carried out in future to assess if this is the case for FIBs as well. This is especially important considering the rise in different ion species, which are becoming more popular now, especially the lighter ion species where the assumption of a flat disc may not hold. Allen et al. showed that He ions can be beneficial when working with thin films [28] as they mostly transmit through the sample. The ion beam-induced heat for various ion species in bulk as well as thin samples (where ions might transmit through the sample) should be systematically investigated and evaluated with 1D and 3D models. The proposed further studies will help develop faster and more robust approaches, especially with the increase in biological and thermally low conductive materials that are processed by FIBs today.

## Experimental

### Collagen sample preparation

Preparation of porcine collagen (UAEC no.1700000190) followed standard methods, as previously described [17]. Briefly, skin was degreased and digested in acetic acid by pepsin. Solubilized collagen was purified by NaCl precipitation at acidic and then neutral pH. Collagen sponges were prepared using purified type-I collagen, glutaraldehyde fixation, and freeze drying, also as previously described [17].

#### Stopping and range of ions in matter (SRIM) simulations of 5 keV gallium ions in skin

The program SRIM (Monte Carlo simulation) was used to determine the heat flux that 5 keV gallium ions (Ga^+^) induce in skin. 50000 ions were simulated for each incident ion energy. The methods “surface sputtering/monolayer collision steps” was selected as the calculation type. The plotting window size was selected in such a way that all ions trajectories in the simulation were completely followed. The calculation parameter output was evaluated to determine the energy losses of each ion for the different interactions. SRIM combines the values for heat and energy required for secondary electron creation in the ionization output value. Therefore, the exact contribution to heat in the inelastic losses (ionization) cannot be determined from the program directly. The presented value will overestimate the energy loss to heat when including the inelastic losses. The following values were used to describe collagen: specific heat *c*_p_ = 3540 J·kg^−1^·K^−1^, density ρ = 1079 kg·m^−3^, thermal conductivity *k* = 0.29 W·m^−1^·K^−1^, and thermal diffusivity α = 1.36 × 10^−7^ m^2^·s^−1^. The SRIM determined heat flux is later used as input parameter for the COMSOL simulations and the numerical modelling of the heat equation.

#### COMSOL simulations for 5 keV gallium ions in collagen

The finite element simulator COMSOL was used to simulate the interaction of 5 keV Ga^+^ with collagen using the time-dependent differential equation of heat conduction for a stationary, homogeneous, and isotropic solid,


[2]

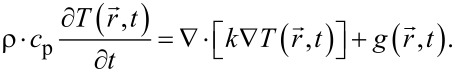



The time evolution of a single gallium ion’s energy deposition into collagen due to the ion’s electronic and nuclear stopping was studied using COMSOL “Particle Tracing Physics”. The dissipated ion energy is converted to thermal energy at each mesh element encountered along the ion’s path in the collagen. For each of these mesh elements the “Heat Transfer Physics’ is used to distribute the resultant thermal energy throughout the rest of the sample. The spatial and temporal evolution of the ions and their heat trail are stored and reassembled after the “Solve” is completed to generate various “Results” plots and images of the sample history.

A 20 nm collagen cube for the 5 keV incident gallium ion case study was chosen, which is based on the ions’ projected range in the collagen material and on a volume representative of the lamella that might ultimately be fabricated in a FIB. The geometry was constrained to room temperature on all sides except at the sample top, which was thermally insulated, as in a vacuum. As the ion passed through the sample, the energy gained by the sample due to the traversing ion’s electronic and nuclear stopping was accumulated. This deposited energy was then conducted away as heat. This heat was distributed to all mesh elements in the vicinity of the ion’s path based on the density, thermal conductivity, and specific heat of collagen.

#### 3D numerical solving of the heat equation by the forward-time central-space method

The time-dependent differential heat equation (Equation 2) was furthermore solved via a numerical modelling approach. To simulate the heat accumulation of multiple ion impacts occurring within a time frame of several nanoseconds, Python was used to implement a forward time–centered space method as a finite-difference method for three dimensions, similar to [29–30].

According to a von Neumann stability analysis [31–32], the differential time step has been calculated to Δ*t* ≤ Δ*x*^2^/(8α) = 23 ps. A sample volume of 600 nm × 600 nm × 400 nm (depth) has been simulated with a voxel size of Δ*x* = 5 nm and the boundary voxels, with exception of the sample surface, have been fixed to room temperature. This approach was chosen since no significant heat dissipation from the surface is expected into the vacuum. Ion impacts are generated in equal time intervals as specified by the average time between two ion impacts and are randomly placed with a uniform spatial distribution on a circular beam profile (specified as the diameter that was used in the experiments). The ion track was simulated by a 10 nm × 10 nm × 20 nm cuboid. A heat input of 0.9·5 keV for each ion was used, which corresponds to the total heat loss of the primary ion. This input parameter was determined via the SRIM simulations. The simulation results have been stored with millikelvin precision in timesteps of 0.1 ns and have been post processed with ImageJ [33]. The temperature rise was calculated according to the volume, density, and specific heat of collagen.

#### FIB cross-sectioning: 5 keV gallium ions in collagen

Multiple cross-sections (15 µm × 10 µm × 200 nm) were cut into the non-resin embedded collagen sample using the FEI Quanta 200 3D at the Queensland University of Technology, Brisbane, Australia. All cross-sections were processed using 5 keV ions. One cross-section was cut with 1.4 nA, 50% overlap to assess if reducing the ion energy is sufficient to avoid heat damage when processing using nA beam currents. 1.4 nA was the highest workable ion beam current at 5 keV ion energy for the device. To further assess the effect of the beam current on sample heating, cross-sections were cut using 0.12 nA, 70 pA as well as 29 pA. For this set of experiments a 20% overlap and 50 nm blur were required to allow patterning with 29 pA and 70 pA to avoid an excessive amount of points which could not be computed by the Quanta 200 3D. The blur was chosen to be smaller than the width of the interaction volume, so that the latter remained the larger parameter and to allow comparison to the previous study. The effect of a blur/overlap combination was evaluated in a second set of experiments by cutting cross-sections using 0.12 nA, 70 pA as well as 29 pA with 20% overlap and 200 nm blur. The beam diameter for 5 keV and 1.4 nA, which is given as 246 nm, exceeds the 200 nm blur and an additional measurement is therefore not meaningful here. The blur was achieved by overfocusing the beam. All cross-sections were prepared using 1 µs dwell time.

SEM images of the prepared cross-sections were recorded with 5 kV acceleration voltage, 90 pA beam current, 1 µs dwell time, 128 frames integration filter and 1024 × 882 pixel resolution with an Everhart–Thornley detector.

#### FIB TEM-lamella preparation and TEM analysis

To verify if the collagen was heat-damaged when processing the sample using nanoampere range currents and different incident ion energies, TEM lamellae for 5 keV ion energy was prepared using the Quanta 200 3D and compared to the previously reported results [17].

First, a protective platinum layer was deposited onto the desired area using 5 kV and 0.23 nA. The cross sections were cut using 5 kV acceleration voltage with 1.4 nA ion beam current, 50% beam overlap. The lifted-out lamella was pre-thinned at 50.5° (front side) and 53.5° (backside) using 5 kV, 0.6 nA, and 50% beam overlap. The final thinning was performed using 5 kV, 0.23 nA, and 50% overlap at 50.5° (front side) and 53.5° (backside). Polishing was not performed as the thinning was already performed with 5 kV acceleration voltage.

The FIB-prepared TEM lamella were analyzed using a Jeol 2100 TEM operating at an acceleration voltage of 200 kV.

## Data Availability

All data that supports the findings of this study is available in the published article and/or the supporting information to this article.
